# Different Combinations of Nitrogen and Carbon Sources Influence the Growth and Postbiotic Metabolite Characteristics of *Lactiplantibacillus plantarum* Strains Isolated from Malaysian Foods

**DOI:** 10.3390/foods13193123

**Published:** 2024-09-30

**Authors:** Qinri Zheng, Suet Lin Chia, Norazalina Saad, Adelene Ai-Lian Song, Teck Chwen Loh, Hooi Ling Foo

**Affiliations:** 1 Department of Bioprocess Technology, Faculty of Biotechnology and Biomolecular Sciences, Universiti Putra Malaysia, 43400 UPM Serdang, Selangor, Malaysia; 2464782691zx@gmail.com; 2 UPM-MAKNA Cancer Research Laboratory (CANRES), Institute of Bioscience, Universiti Putra Malaysia, 43400 UPM Serdang, Selangor, Malaysia; suetlin@upm.edu.my (S.L.C.); norazalina@upm.edu.my (N.S.); 3 Department of Microbiology, Faculty of Biotechnology and Biomolecular Sciences, Universiti Putra Malaysia, 43400 UPM Serdang, Selangor, Malaysia; adelene@upm.edu.my; 4 Department of Animal Science, Faculty of Agriculture, Universiti Putra Malaysia, 43400 UPM Serdang, Selangor, Malaysia; tcloh@upm.edu.my; 5 Research Laboratory of Probiotics and Cancer Therapeutics, UPM-MAKNA Cancer Research Laboratory (CANRES), Institute of Bioscience, Universiti Putra Malaysia, 43400 UPM Serdang, Selangor, Malaysia

**Keywords:** *Lactiplantibacillus plantarum*, postbiotic, antioxidant activity, lactic acid, inhibitory activity

## Abstract

Postbiotic metabolites produced by *Lactiplantibacillus plantarum* strains isolated from Malaysian food have been extensively reported for their positive effects on health. Understanding the effects of different combinations of carbon and nitrogen sources on the growth and corresponding characteristics of postbiotic metabolites produced by different strains of *L. plantarum* is important for various applications. Hence, the effects of different combinations of carbon (glucose, lactose, sucrose and dextrose) and nitrogen (X-SEED Kat, X-SEED Peptone, X-SEED Nucleo Advanced, Nucel875 MG, FM888 and FM902) sources on the growth of six strains of *L. plantarum* (RG11, RG14, RI11, RS5, TL1 and UL4) and the functional characteristics (bacteriocin inhibitory activity, antioxidant activity and lactic acid concentration) of their respective postbiotic metabolites were investigated in this study. UL4 produced the highest viable cell population with sucrose and Nucel875 nitrogen source. The UL4 strain also produced the strongest bacteriocin inhibitory activity with dextrose and FM888 nitrogen source. In comparison, the RI11 strain produced the highest lactic acid concentration with dextrose and Nucel875 nitrogen source and the highest reducing power of RS5 and TL1 postbiotic metabolites was achieved with MRS medium. In the combination of sucrose and X-Seed KAT nitrogen source, RG14 produce the highest hydroxyl radical scavenging activity. The effects of different combinations of carbon and nitrogen sources on the viable cell population of *L. plantarum* strains and the respective functional characteristics of postbiotic metabolites were strain dependent. The current study also revealed that fermentation media were an important factor that greatly impacted the functionalities of postbiotic metabolites due to the presence of various bioactive compounds that contributed to high antioxidant and antimicrobial properties. The results of this study will facilitate the subsequent medium design and optimisation for the development and production of specific postbiotic metabolites produced by the respective *L*. *plantarum* strain for their applications in various industries.

## 1. Introduction

*Lactiplantibacillus plantarum* is a dominant species of lactic acid bacteria (LAB) which has played an important role in food fermentation for centuries. LAB have been widely accepted as harmless microorganisms for years, attributed to their natural inhibitory characteristics [1,2,3,4,5]. The food fermented by *L. plantarum* has a unique flavour [6,7], possibly due to various metabolites produced during fermentation [8,9]. Previously, we have isolated and characterised a few *L. plantarum* strains from Malaysian foods, such as steamed fish [10], fermented durian pulp [11], fermented tapioca [12] and fermented fish sauce [13]. We have also extensively reported the myriad functionalities and health impacts of postbiotic metabolites produced by the six strains of *L. plantarum* (RG11, RG14, RI11, RS5, TL1 and UL4) isolated from Malaysian foods. Their postbiotic metabolites exhibited broad inhibitory activity against various pathogens [2,14] and induced cytotoxicity against various cancer cells [15,16]. In addition, the postbiotic metabolites produced by these *L. plantarum* strains have been proven as promising growth promoters for broiler chickens [17,18,19], laying hens [20,21,22,23], rats [24,25,26,27], postweaning piglets [28,29] and postweaning lambs [30,31]. Postbiotics offer physiological benefits to the animal host by suppressing the gut pathogen populations and improving the mucosal gut barrier integrity [17,18,19].

Tsilingiri and Rescigno [32,33] defined postbiotics as non-viable soluble bioactive metabolites produced by probiotics. Different fermentation processes, such as growth medium and physical parameters, affect the composition of soluble bioactive components of postbiotics [34,35,36,37]. The bioactive components of postbiotics comprise low-molecular-mass compounds, such as organic acids, predominantly lactic acid, bacteriocins, diacetyl, acetaldehyde, and others [8,9]. 

New evidence now suggests that probiotic beneficial effects may not necessarily be mediated through the live bacteria cells; instead, they are mediated via the active soluble bioactive compounds known as postbiotic metabolites produced by the live probiotic cell. Thus, probiotic effects could be based on the viability of probiotic cells, soluble metabolites, or probiotic cell lysis products [32,33,38]. Postbiotics are easier to keep and transport, as a low temperature for storage is not required. Postbiotics do not contain live microorganisms; hence, the risk of ingesting postbiotics is fairly low [39]. In the food industry, the bacteriocin of postbiotic metabolites has been used as a natural bio-preservative in dairy products [40]. In agricultural applications, postbiotics have been proposed as a substitute for anti-microbial growth promoters (AGP) to enhance the growth of poultry [18,19,20,21,22,23]. In addition, postbiotics infeed supplementation promote animal development [22,25] and improve blood antioxidant activity and immune response [17,18,19]. Previous research evidence has shown that postbiotics can enhance intestinal health by secreting a wide range of compounds, including organic acids, nutrients, their enzymes, extracellular proteins, indoles, immune signalling compounds, cofactors, and substances that regulate the intestinal epithelial cell barrier [41,42]. Furthermore, postbiotics’ anti-microbial peptide bacteriocin molecules function as anti-microbial agents against various pathogens, including *Salmonella* spp. and *Escherichia coli* [43]. 

An ideal media is essential to provide carbon, nitrogen, and other nutrients [44] for the growth, metabolic activity, and production of specific metabolites by producer cells. As for postbiotic metabolite production by *L. platanrum* strains, much attention has been given to fermentation technology development and media optimisation [34,35,36,37]. Medium composition is an important parameter affecting microorganisms’ growth during fermentation. Depending on their nutrient requirements, microorganisms need different sources of carbon, nitrogen, minerals, and vitamins. Improper medium composition may impede cell growth and metabolic activity [45]. Glucose was previously considered the main carbon source in most studies; however, *Enterococcus faecium* exhibits a distinct sucrose fermentation mode [46]. In addition, *L. plantarum* RS5 can utilise various carbon sources for bacteriocin production [34,35,36,37]. The optimal concentration of carbon and nitrogen sources is crucial for producing postbiotic metabolites [47]. 

Although postbiotic metabolites have been extensively proven to be promising growth enhancers and health supplements in many research articles, the effects of different combinations of carbon and nitrogen sources on cell growth and respective postbiotic metabolite characteristics have not been explored. Therefore, this study aims to determine the impact of different combinations of carbon and nitrogen sources on cell growth and respective postbiotic metabolite characteristics, such as antioxidant activity, bacteriocin inhibitory activity, and lactate concentration, produced by the six strains of *L. plantarum* isolated from Malaysian foods. The best combination of carbon and nitrogen sources will facilitate the production of postbiotic metabolites for respective industrial applications.

## 2. Materials and Methods

### 2.1. Materials

Carbon sources: glucose, sucrose, lactose, and dextrose were purchased from Merck (Darmstadt, Germany). Nitrogen sources: X-SEED KAT, X-SEED Peptone and X-SEED Nucleo Advanced, yeast extracts were obtained from Ohly yeast extracts (Hamburg, Germany), Nucel875 MG yeast extract was purchased from Procelys (Alfort Cedex, France), whereas FM888 and FM902 were obtained from Angle Yeast (Yichang, China).

### 2.2. Bacterial Cultures and Maintenance

Six *L. plantarum* strains, RG11, RG14, RI11, RS5, TL1, and UL4, were previously isolated from Malaysian fermented foods [10,12] and obtained from the Laboratory of Industrial Biotechnology, Department of Biotechnology, Faculty of Biotechnology and Biomolecular Sciences, Universiti Putra Malaysia. The *L. plantarum* strains and *Pediococcus acidilactici* ATCC 4-46 were revived and maintained according to the previously described methods [12,34,35,36,37] using de Man, Rogosa, and Sharpe (MRS) medium (Merck, Darmstadt, Germany).

### 2.3. Postbiotic Metabolite Preparation

A 1% (*v*/*v*) volume of active *L. plantarum* strains was inoculated into 10 mL MRS medium and incubated at 37 °C for 24 h. The active cell pellets were collected by centrifugation at 10,000× *g* for 15 min at 4 °C, followed by washing the cell pellets 3 times with 0.85% (*w*/*v*) NaCl (Merck, Darmstadt, Germany) solution and, adjusting them to an optical density of 1 to 1.5 at 600 nm using a UV–visible spectrophotometer (Agilent Technologies, Santa Clara, CA, USA), which corresponded to 10^9^ cfu/mL to be used as an active inoculum. Table 1 shows the medium compositions used for producing respective postbiotic metabolites by the 6 strains of *L. plantarum*. 

The MRS medium was employed as the control medium. An amount of 1% (*v*/*v*) of the adjusted *L. plantarum* strain was inoculated into 10 mL respective medium and incubated at 37 °C for 24 h, followed by centrifugation (10,000× *g* for 15 min at 4 °C) to separate the bacterial biomass from the fermented medium. The bacterial cell pellet was collected for bacterial viable cell determination, where the fermented medium was filtered through a 0.2 µm cellulose acetate membrane (Sartorius Stedim, Germany), and the filtrate was designated as a postbiotic metabolite and kept at −20 °C for the determination of bacteriocin inhibitory activity, lactic acid concentration, and antioxidant activities. The postbiotic metabolite preparation was conducted in triplicate.

### 2.4. Bacterial Viable Cell Determination

The bacterial cell population was determined using the total plate count method [26,48]. In brief, 10-fold dilutions (10^0^ to 10^−9^) were made with 0.85% (*w*/*v*) NaCl solution for the bacterial cell pellets collected from Section 2.3. The viable cell determination was conducted by spreading 50 μL of the respective diluted cell suspension (10^−6^ to 10^−8^) onto MRS agar (Merck, Darmstadt, Germany) and incubating at 30 °C for 48 h. The cell viability determination was conducted in triplicate.

### 2.5. Bacterioicn Inhibitory Activity Determination 

According to the procedure described by Ooi et al. [36], the bacteriocin inhibitory activity of the postbiotic metabolites was determined by the modified agar well diffusion method [12,49]. A two-fold serial dilution was conducted to dilute postbiotic metabolites using a sterile 0.85% (*w*/*v*) NaCl solution. A volume of 20 µL diluted postbiotic metabolites (2^0^ to 2^−5^) was then allowed to diffuse in a pre-punched MRS agar well (5.0 mm in diameter) before overlaying with 3 mL of soft agar inoculated with 1% (*v*/*v*) *P. acidilactici* ATCC 4–46 (OD_600nm_ was adjusted to 1.0) and incubating at 30 °C for 48 h. A clear inhibition zone with a diameter of more than 1 cm (including the 0.5 cm diameter of the well) was considered a positive bacteriocin inhibitory activity. The bacteriocin inhibitory activity was determined in triplicate and expressed as the modified arbitrary unit (MAU/mL), as shown below [12]:

$$\begin{array}{l} \mathrm{Modified}\;\mathrm{arbitrary}\;\mathrm{unit}\;[\mathrm{MAU}/\mathrm{mL}]\;=\;\\ \frac{\mathrm{Reciprocal}\;\mathrm{of}\;\mathrm{the}\;\mathrm{highest}\;\mathrm{dilution}\;\mathrm{factor}\;\mathrm{yielded}\;\mathrm{clear}\;\mathrm{zone}\;[\mathrm{AU}]}{\mathrm{Volume}\;\mathrm{of}\;\mathrm{postbiotic}\;\mathrm{generated}\;\mathrm{positive}\;\mathrm{antimicrobial}\;\mathrm{activity}\;[\mathrm{mL}]}\;\times \;\mathrm{Clear}\;\mathrm{zone}\;\mathrm{diameter}\;[\mathrm{cm}] \end{array}$$

### 2.6. Lactic Acid Concentration Determination 

The procedure of Borshchevskaya et al. [50] was employed to determine the lactic acid concentration (g/L) in the postbiotic metabolites collected in Section 2.3. Appropriately diluted postbiotic metabolites were added with 2 mL 0.2% (*w*/*v*) iron (III) chloride, followed by reading the optical density at 390 nm using a UV–visible spectrophotometer (Agilent Technologies, USA) within 15 min of mixing. Lactic acid solutions (L6661, Sigma Chemical, St. Louis, MO, USA) at the concentration of 0–80 mg/mL was used to construct the lactic acid standard curve. The determination of lactic acid concentration was conducted in triplicate.

### 2.7. Antioxidant Activity Determination

Hydroxyl radical scavenging (HRS) and reducing power (RP) assays were performed to determine the antioxidant activities of the postbiotic metabolites collected in Section 2.3 according to the methods described by Xing et al. [51] with slight modifications.

#### 2.7.1. Reducing Power Assay

A volume of 250 μL of the postbiotic metabolite sample was mixed with 250 μL of phosphate buffer (0.2 M, pH 6.6) and 250 μL of 1% (*w*/*v*) potassium ferricyanide, followed by heating in a 50 °C water bath for 20 min. The assay mixture solution was then cooled down to room temperature before adding 250 μL 10% (*w*/*v*) trichloroacetic acid. The precipitate was removed by centrifugation at 3000× *g* for 5 min at 4 °C. The supernatant was collected and added with 250 μL deionised water and 500 μL 0.1% (*w*/*v*) iron (III) chloride before absorbance was taken at 700 nm. Ascorbic acid was used as the standard reference. The RP assay was conducted in triplicate.

#### 2.7.2. Hydroxyl Radical Scavenging Assay

In brief, the 250 µL sample was mixed with 250 μL of 2.5 mM 1,10-phenanthroline, 250 μL of phosphate buffer (0.1 M, pH 7.4), and 250 μL of 2.5 mM FeSO4, followed by mixing with 250 μL of 20 mM H_2_O_2_. The assay mixture was then incubated at 37 °C for 90 min. A UV–visible spectrophotometer (Agilent Technologies, USA) was used to measure the absorbance at 536 nm. The HRS activity was calculated based on the following equation:

$$HRS\;activity\;(\%)=(A_{s}-A_{c})/(A_{b}-A_{c})\times 100\%$$

A_s_ is the absorbance of the postbiotic metabolite sample solution, A_c_ is the absorbance containing the deionised water, and A_b_ is the absorbance of the reagent mixture without the presence of the postbiotic metabolite sample and H_2_O_2_. The HRS assays were conducted in triplicate.

### 2.8. Statistical Analysis

The data are presented as mean and standard deviation. The collected data were analysed using SPSS (version 26.0, IBM Inc., Chicago, IL, USA) for one-way analysis of variance (ANOVA) with the Tukey test to compare a significant difference in means at *p* < 0.05 with the respective result obtained from the MRS medium. A correlation analysis was conducted to determine the relationship between the viable cell count of the *L. plantarum* strain and the functional characteristics of the postbiotic metabolite.

## 3. Results and Discussion

### 3.1. Bacterial Viable Cell Population

**Figure 1:**
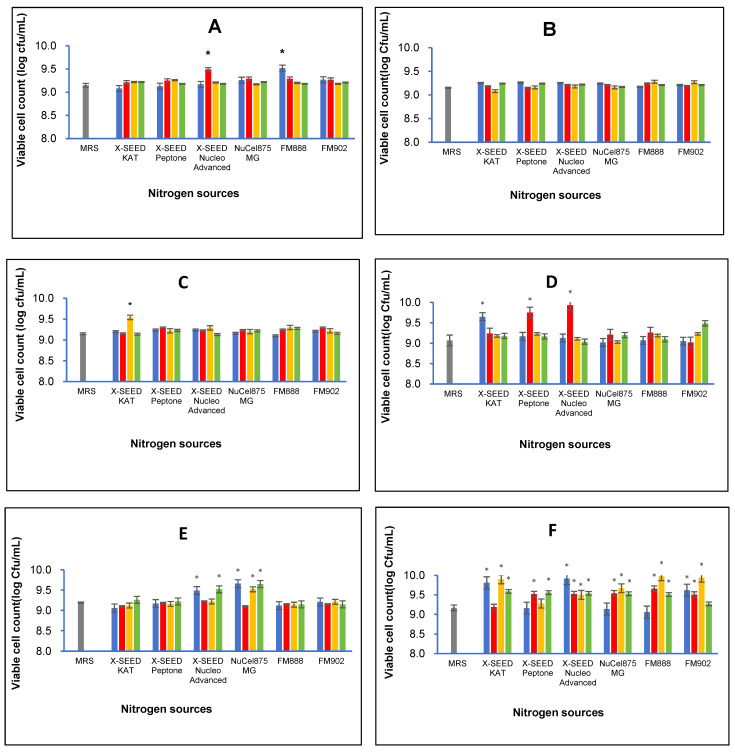
Effects of different combinations of carbon and nitrogen sources on viable cell number of six *Lactiplantibacillus plantarum* strains. (**A**) RG11; (**B**) RG14; (**C**) RI11; (**D**) RS5; (**E**) TL1; and (**F**) UL4 strains. MRS medium was used as a control medium. The values for viable cell number are the mean ± standard deviation (SD), n = 3. The asterisk indicates a significant difference (*p* < 0.05). 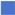 Glucose, 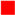 Lactose, 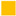 Sucrose and 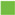 Dextrose.

The combination of different carbon and nitrogen sources significantly affected the cell growth of the six *L. plantarum* strains. Generally, Figure 1 illustrates that the best combination of nitrogen and carbon sources differs amongst the strains. Table 2 summarises the highest viable cell populations of the six strains of *L. plantarum* exerted by the best combination of specific carbon and nitrogen sources. As seen from Figure 1 and Table 2, the RG11 strain produced the significantly highest number of viable cells (log 9.52 cfu/mL, *p* < 0.05) when grown in a medium containing glucose as a carbon source and FM888 as a nitrogen source. However, the highest cell growth (log 9.28 cfu/mL) of the RG14 strain was observed from a growth medium comprising sucrose as a carbon source and FM888 as a nitrogen source. The RI11 strain showed the highest cell growth activity of log 9.54 cfu/mL (*p* < 0.05) via sucrose as a carbon source and X-SEED KAT as a nitrogen source. In contrast, the RS5 strain produced the highest cell growth activity of log 9.93 cfu/mL (*p* < 0.05) when lactose was used as a carbon source and X-SEED Nucleo Advanced was used as a nitrogen source. As for the TL1 strain, the highest viable cell number of log 9.66 cfu/mL (*p* < 0.05) was detected via dextrose as a carbon source and Nucel875 MG as a nitrogen source. The UL4 produced the highest cell growth activity (log 9.98 cfu/mL, *p* < 0.05) amongst the *L. plantarum* strains when it grew in a growth medium comprising sucrose as a carbon source and Nucel875 MG as a nitrogen source.

The results obtained in this experiment agreed with previous experiments, whereby the six strains of *L. plantarum* grew well in all formulated media with cell counts of more than 9 log cfu/mL. Ooi et al. [36] reported the effects of carbon and KAT yeast extract nitrogen sources on the bacteriocin inhibitory activity of postbiotic metabolites and the respective growth of the *L. plantarum* I-UL4 strain. The dry cell weight of the UL4 strain increased along with the concentration of yeast extract supplemented in the growth medium. Subsequently, they optimised a refined medium via statistical-based approaches (Fractional Factorial Design and Central Composite Design of Response Surface Methodology) to enhance the antimicrobial activity of postbiotic metabolites and the growth of the *L. plantarum* RS5 strain [24]. The significantly highest (*p* < 0.05) biomass of 3.41 g/L (24 h) of the RS5 strain was attained in the growth medium containing 44.55 g/L of X-SEED KAT yeast extract.

In addition, Mohamad et al. [48] enhanced the versatile extracellular cellulolytic and hemicellulolytic enzyme and the growth of the *L. plantarum* RI 11 strain using yeast extract and other renewable natural polymers. They demonstrated similar growth profiles of the RI 11 strain in media supplemented with rice straw and yeast extract, molasses and yeast extract, and PKC and soybean pulp, respectively. The variability in nutrient preferences highlights the importance of optimising specific carbon and nitrogen sources [34,36] to enhance the growth and productivity of each *L. plantarum* strain employed in this study. By tailoring nutrient composition, such as carbon and nitrogen sources, postbiotic metabolite production can be enhanced effectively, potentially leading to higher yields and better quality via a defined fermentation process [34,35,36,37].

### 3.2. Bacterioicn Inhibitory Activity 

**Figure 2:**
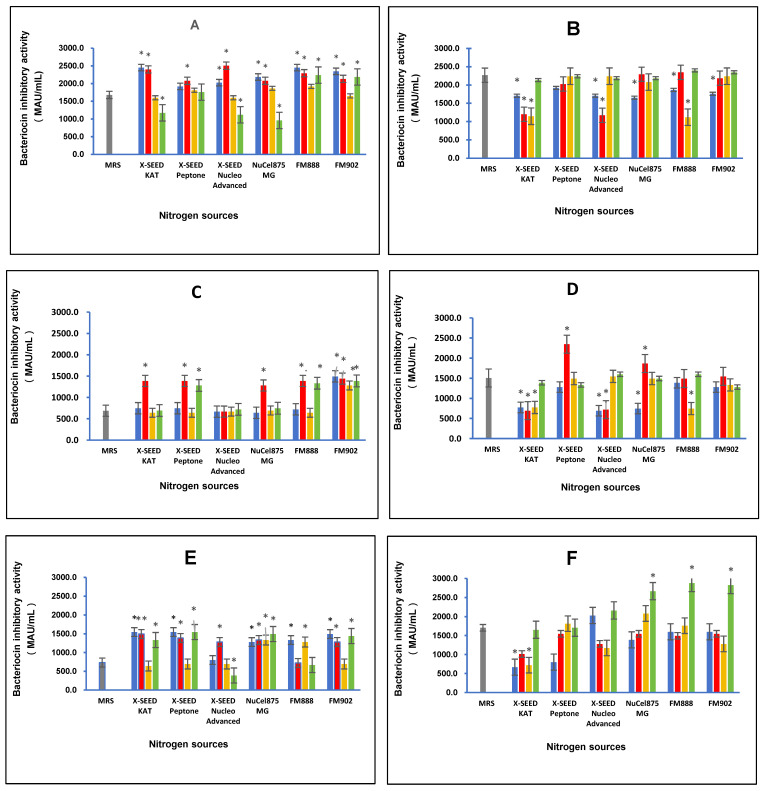
Effects of different combinations of carbon and nitrogen sources on the bacteriocin inhibitory activity of six *Lactiplantibacillus plantarum* strains. (**A**) RG11; (**B**) RG14; (**C**) RI11; (**D**) RS5; (**E**) TL1; and (**F**) UL4 strains. MRS medium was used as a control medium. The values for bacteriocin-inhibitory activity are the mean ± standard deviation (SD), n = 3. The asterisk indicates a significant difference (*p* < 0.05). 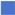 Glucose, 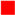 Lactose, 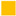 Surcose and 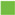 Dextose.

The effect of different combinations of carbon and nitrogen sources on the bacteriocin inhibitory activity of postbiotic metabolites produced by the six *L. plantarum* strains is illustrated in Figure 2. The different combinations of carbon and nitrogen sources significantly (*p* < 0.05) affected the bacteriocin inhibitory activity of the postbiotic metabolites produced by the six *L. plantarum* strains against the indicator *P. acidilactici* ATCC 4–46. The highest bacteriocin inhibitory activity of 2506.67 MAU/mL was detected for RG11 postbiotic metabolites when the growth medium comprised lactose as the carbon source and X-SEED Nucleo Advanced as the nitrogen source. However, for the postbiotic metabolite RG14, the growth medium comprising dextrose as the carbon source and FM888 as the nitrogen source induced the highest bacteriocin inhibitory activity of 2293.33 MAU/mL. The highest bacteriocin inhibitory activity of RI11 postbiotic metabolites of 1493.33 MAU/mL was noted when glucose as the carbon source and FM902 as the nitrogen source were supplemented in the growth medium. Nevertheless, when the growth medium consisted of lactose as the carbon source and X-SEED Peptone as the nitrogen source, the RS5 strain produced the highest bacteriocin inhibitory activity of 2346.67 MAU/mL. As for the TL1 strain, the growth medium consisting of glucose as the carbon source and X-SEED KAT as the nitrogen source induced the highest bacteriocin inhibitory activity of 1546.67 MAU/mL. In comparison, the UL4 strain produced the strongest bacteriocin inhibitory activity of 2880 MAU/mL amongst the six *L. plantarum* strains when the growth medium comprised dextrose as the carbon source and FM888 as the nitrogen source. 

In this experiment, the postbiotic metabolites produced by the six strains of *L. plantarum* demonstrated significant (*p* < 0.05) different bacteriocin inhibitory activity when grown in a medium containing different combinations of carbon and nitrogen sources, which was consistent with the bacteriocin inhibitory of the *L. plantarum* strains reported previously [14,43]. The *L. plantarum* strains employed in this study have been reported to harbour two classes of bacteriocin genes, namely, *planEF* and *planW*, which are responsible for the production of plantaricin Ef and plantaricin W bacteriocins, respectively [4,52,53,54]. The composition of the culture medium is one of the important elements that has been shown to greatly affect bacteriocin production [34,35,36,37]. Therefore, in this experiment, the effect of different combinations of carbon sources (glucose, lactose, sucrose, and dextrose) and nitrogen sources (X-SEED Kat, X-SEED Peptone, X-SEED Nucleo Advanced, Nucel875 MG, FM888 and FM902), on the bacteriocin inhibitory activity of postbiotic metabolites produced by the six *L. plantraum* strains was verified further to confirm the best combination of carbon and nitrogen sources for each *L. plantarum* strain for the production of postbiotic metabolites exhibiting the highest bacteriocin inhibitory activity. The information on the best combination of carbon and nitrogen sources for the highest bacteriocin inhibitory activity of each *L. plantarum* strain is essential for the effective production of a postbiotic metabolite to be used as a potential bio-preservative in the food industry or as a promising substitute for the in-feed antibiotic growth promoter of the livestock industry. The results of this study imply that the specific combinations of carbon and nitrogen sources play a crucial role in the bacteriocin inhibitory activity of postbiotic metabolites produced by *L. plantarum* strains employed in this study, contributing to the significant development and production of more effective probiotics and postbiotic metabolites [38,42] tailored to target specific pathogens [8,9,55,56] and various respective applications.

### 3.3. Lactic Acid Concentration

**Figure 3:**
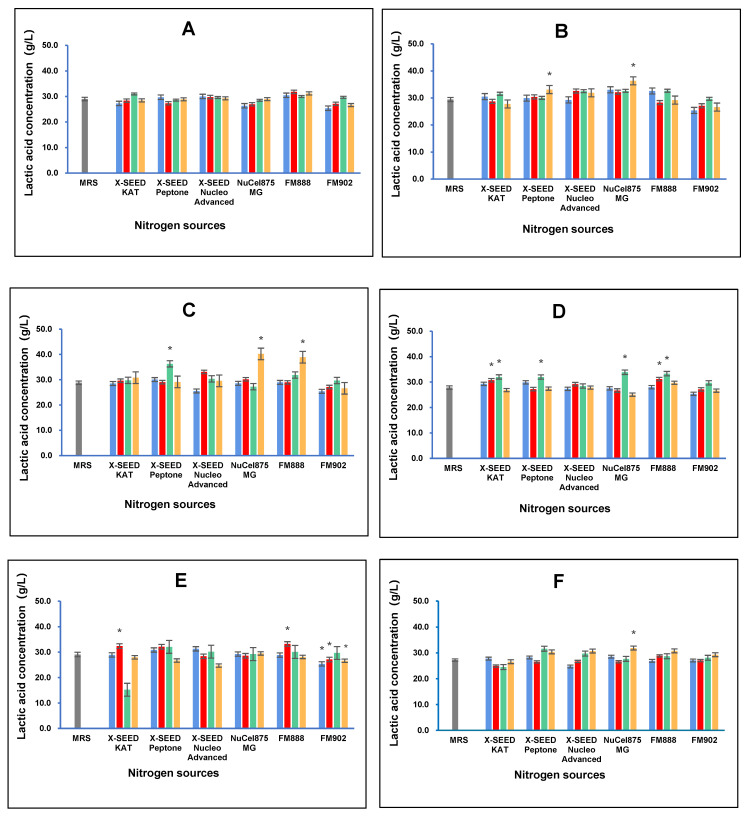
Effects of different combinations of carbon and nitrogen sources on the lactic acid concentration produced by six *Lactiplantibacillus plantarum* strains. (**A**) RG11; (**B**) RG14; (**C**) RI11; (**D**) RS5; (**E**) TL1; and (**F**) UL4 strains. MRS medium was used as a control medium. The results of lactic acid concentrations are the mean ± standard deviation (SD), n = 3. The asterisk indicates a significant difference (*p* < 0.05). 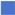 Glucose, 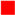 Lactose, 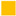 Surcose and 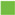 Dextrose.

The effect of different carbon and nitrogen sources on the lactic acid concentration of postbiotic metabolites produced by the six *L. plantarum* strains is shown in Figure 3. The results demonstrated that the combination of different nitrogen and carbon sources has different effects on lactic acid production by the six *L. plantarum* strains. The RG14 strain produced the highest lactic acid concentration (36.34 g/L) when lactose was used as the carbon source and FM888 as the nitrogen source. The RI11 strain generated the greatest lactic acid concentration (40.21 g/L) when grown in a medium containing dextrose as the carbon source and Nucel875 MG as the nitrogen source. In contrast, the RS5 strain produced the most lactic acid (33.85 g/L) concentration when dextrose was used as the carbon source and Nucel875 MG as the nitrogen source. However, TL5 produced the greatest lactic acid concentration (33.21 g/L) when sucrose was the carbon source and Nucel875 MG was the nitrogen source. As for the UL4 strain, the highest lactic acid concentration (31.85 g/L) was obtained when dextrose was the carbon source and Nucel875 MG was the nitrogen source. In comparison, the RI11 strain produced the highest lactic acid concentration amongst the six strains, with dextrose as the carbon source and Nucel875 MG as the nitrogen source. Interestingly, the different combinations of carbon and nitrogen sources have little effect on lactic acid production by the RG11 strain. 

All *L. plantarum* strains employed in this study produced lactic acid during growth, as reported by Van Thu et al. [43], a predominant characteristic of LAB [57]. The choice of carbon and nitrogen sources is crucial because they are essential nutrients that influence the growth of LAB cells and metabolic activities [58]. Different carbon and nitrogen sources influence lactic acid production by LAB, attributing to varying amounts of energy and building block supplies for growth and metabolic activities [59]. Chang et al. [8] reported that the RS5 strain produced the highest (*p* < 0.05) concentration of lactic acid when grown in MRS and a formulated medium containing glucose as the carbon source and X-SEED KAT as the nitrogen source. Lactic acid produced by LAB is an important food additive that functions as generally recognised as safe (GRAS) natural antimicrobial agent [60], inhibiting food spoilage bacteria, such as Gram-negative bacteria of the *Enterobacteriaceae* family and Gram-positive *Listeria monocytogenes* [8]. In addition, lactic acid has been reported to have many applications. For example, lactic acid can lower gut pH and disrupt bacterial nutrient uptake and energy absorption by lysing bacterial cell membranes [55,56,57], reducing the risk of pathogen infection [61,62].

### 3.4. Antioxidant Activities

A growing number of studies show that antioxidation is essential to enhancing human health [63]. Excessive oxidative stress can induce inflammation, cellular damage, and cancer development [64]. Previous studies have shown that the postbiotic metabolites produced by *L. plantarum* B1–6 can primarily reduce protein and lipid oxidation through the antioxidant activities of hydroxyl radical scavenging and reducing power activities [65]. In addition, the antioxidant capacity of *L. plantarum* postbiotic metabolites could also play an important role in cancer prevention and treatment [15,16].

#### 3.4.1. Reducing Power Determination

**Figure 4:**
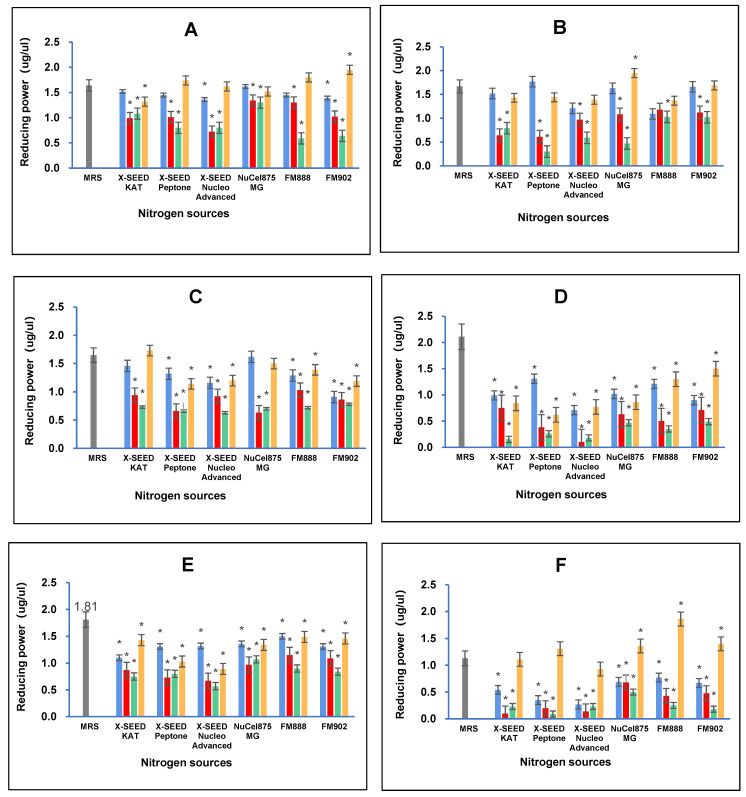
Effects of different combinations of carbon and nitrogen sources on the reducing power of postbiotic metabolites produced by the six *Lactiplantibacillus plantarum* strains. (**A**) RG11; (**B**) RG14; (**C**) RI11; (**D**) RS5; (**E**) TL1; and (**F**) UL4 strains. MRS medium was used as a control medium. The values of reducing power are the mean ± standard deviation (SD), n = 3. The asterisk indicates a significant difference (*p* < 0.05). 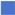 Glucose, 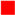 Lactose, 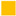 Sucrose and 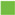 Dextrose.

The results of the reducing power analysis showed that combining different carbon and nitrogen sources greatly impacted the reducing power antioxidant capacity of the postbiotic metabolites produced by the *L. plantarum* strains employed in this study. Figure 4 illustrates that the highest reducing power activity of RG11 postbiotic metabolites (1.95 ug/uL, *p* < 0.05) was detected when RG11 was grown in a medium containing dextrose as the carbon source and FM902 as the nitrogen source. As for the postbiotic metabolites of RG14, the highest reducing power activity of 1.95 ug/uL (*p* < 0.05) was noted when the RG14 strain was grown in a medium comprising dextrose as the carbon source and FM902 as the nitrogen source. The highest reducing power activity for the RI11 postbiotic metabolites (1.73 ug/uL, *p* < 0.05) was obtained when RI11 was grown in a medium consisting of glucose as the carbon source and X-SEED KAT as the nitrogen source. However, the highest reducing power of RS5 and TL1 postbiotic metabolites was detected when both strains were grown in an MRS medium with a reducing power of 2.11 ug/uL (*p* < 0.05) and 1.81 ug/uL (*p* < 0.05), respectively. The highest reducing power activity of UL4 postbiotic metabolites (1.86 ug/uL, *p* < 0.05) was noted when glucose was used as the carbon source and FM888 was used as the nitrogen source.

Comparing the reducing power capacities of the postbiotic metabolites produced by the six strains of *L. planatrum*, it is evident that different carbon and nitrogen sources significantly influence the antioxidant capabilities of postbiotic metabolites. Different strains have specific preferences for carbon and nitrogen sources to produce the highest reducing power capacity. For instance, the postbiotic metabolites of RG11 and RG14 produced the highest reducing power activity when both strains were grown in a medium containing dextrose and FM902, while RI11 excelled with glucose and X-SEED KAT. Interestingly, RS5 and TL1 displayed the highest reducing power activity when MRS was used as the growth medium. Nevertheless, the highest reducing power of UL4 postbiotic metabolites was noted with the combination of glucose and FM888. The results obtained in this study agree with previous reports [8,9,66,67,68]. Reducing power activity is likely to be mediated through the inhibition of the oxidation process by converting hydroperoxides to hydroxyoctadecadienoic acids and iron chelators in postbiotic metabolites, attributed to the presence of various intracellular antioxidants such as pyrrole compounds present in the postbiotic metabolites produced by the *L. plantarum* strains employed in this study [8,9,69]. Moreover, the different reducing power activity exhibited by different postbiotic metabolites produced by using different combinations of carbon and nitrogen sources could be due to the metal ion chelating ability, the antioxidant enzyme system and the antioxidant metabolites present in the postbiotic metabolites produced by the six strains of *L. plantarum* employed in this study [70]. Therefore, postbiotic metabolites have the potential to be a promising supplement and feed additive to treat inflammation induced by oxidative stress-related diseases [64,71].

#### 3.4.2. Hydroxyl Radical Scavenging Activity

**Figure 5:**
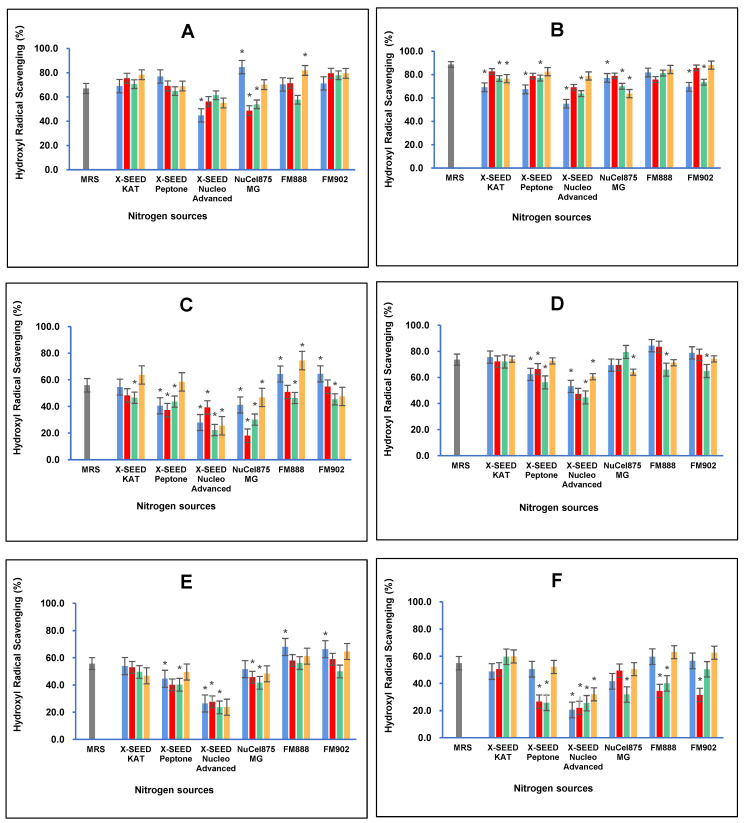
Effects of different combinations of carbon and nitrogen sources on the hydroxyl radical scavenging activity of postbiotic metabolites produced by the six *Lactiplantibacillus plantarum* strains (**A**) RG11; (**B**) RG14; (**C**) RI11; (**D**) RS5; (**E**) TL1; and (**F**) UL4 strains. MRS medium was used as a control medium. The hydroxyl radical scavenging activity values are the mean ± standard deviation (SD), n = 3. The asterisk indicates a significant difference (*p* < 0.05). 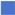 Glucose, 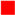 Lactose, 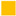 Sucrose and 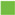 Dextose.

Similarly, carbon and nitrogen sources greatly influence the hydroxyl radical scavenging antioxidant activity of postbiotic metabolites produced by *L. plantarum* strains, as illustrated in Figure 5. The results demonstrated that the highest hydroxyl radical scavenging activity (84.66%, *p* < 0.05) of the postbiotic metabolites of RG11 was detected when the RG11 strain was grown with glucose as the carbon source and Nucel875 MG as the nitrogen source. The combination of sucrose and X-SEED KAT as carbon and nitrogen sources, respectively, induced RG14 to produce the highest hydroxyl radical scavenging activity of 88.65% (*p* < 0.05). The highest hydroxyl radical scavenging of postbiotic metabolite RI11 (74.49%, *p* < 0.05) was obtained when dextrose as the carbon source and FM888 as the nitrogen source were used to grow the RI11 strain. In comparison, for the RS5 strain, the highest hydroxyl radical scavenging activity of RS5 postbiotic metabolites (73.59% *p* < 0.05) was produced with MRS media, which was also noted for the reducing power antioxidant activity. However, the TL1 strain produced the highest hydroxyl radical scavenging activity of 67.95% (*p* < 0.05) using glucose as the carbon source and FM888 as the nitrogen source. The UL4 strain produced the highest hydroxyl radical scavenging activity of 63.05% (*p* < 0.05) using dextrose as the carbon source and FM888 as the nitrogen source. In comparison, the RG14 strain produced the highest hydroxyl radical scavenging activity of 88.65% (*p* < 0.05) amongst the *L. plantarum* strains employed in this study.

These findings suggest that enhancing the antioxidant activity of postbiotic metabolites could be mediated by optimising the carbon and nitrogen sources for *L. plantarum* fermentation. The postbiotic metabolites possess significant antioxidant activities that could warrant broad applications in various industries, particularly in the food and pharmaceutical industries, such as functional foods, supplements, and therapeutic agents. Furthermore, these postbiotics could play a role in improving gut health and reducing oxidative stress-related diseases [72,73]. Natural antioxidant sources have been used to counteract the implications of oxidative stress and reactive oxygen species. Since oxidative stress antagonistically influences inflammation, cellular damage, and disease susceptibility in the body [64], the postbiotic metabolites produced by the *L. plantarum* strains employed in this study have vast potential to reduce the oxidation of proteins and lipids via hydroxyl radical scavenging and reducing power activities. The antioxidant activity exerted by postbiotic metabolites would scavenge and inhibit free radicals from oxidation processes [74]. Chen et al. [74] reported that the fermentation of papaya juice using *L. plantarum* produced higher antioxidant activities than *L. acidophilus*. Furthermore, the in-feed supplementation of *L. plantarum* postbiotic metabolites has been documented to reduce the negative effect stimulated by hepatic injury in mice [75]. Therefore, *L. plantarum* postbiotic metabolites have been extensively proven as a promising natural source of antioxidants to reduce the effects of heat stress in animals [17,72,73].

### 3.5. Correlation between L. plantarum Strain Growth and Postbiotic Metabolite Functional Characteristics

Correlations between the growth of *L. plantarum* strains and the functional characteristics of prosthetic metabolites are shown in Table 3. Interestingly, a significant negative correlation was demonstrated between *L. plantarum* cell growth and antioxidant activity (*p* < 0.05). This negative correlation could be due to the metabolic trade-offs where resources allocated to cell growth might limit the production of antioxidant compounds [62,76,77]. Additionally, a high cell density could lead to nutrient depletion, stressing the cells and suppressing the production of antioxidants [59,65], attributed to the metabolic heterogeneity of *L. plantarum* strains. However, a positive correlation between bacteriocin inhibitory activity and the antioxidant activity of reducing power and hydroxyl radical clearance (*p* < 0.05) was noted, implying that the bacteriocin inhibitory compounds may possess antioxidant properties [65,66,67], which could remove or neutralise free radicals and excessive oxidative stress. Thus, postbiotic metabolites are a promising health or feed supplement for preventing inflammation, cellular damage, and cancer development. Nonetheless, there is no significant correlation between *L. plantarum* cell growth and bacteriocin inhibitory activity or lactic acid concentration. 

Interestingly, there is no significant correlation between lactic acid concentration and *L. plantarum* cell growth, bacteriocin inhibitory activity, or antioxidant activity, suggesting that the *L. plantarum* strains likely use different metabolic pathways for the production of predominant lactic acid, bacteriocin inhibitory and antioxidant compounds. However, in contrast, Ooi et al. [36] reported a significantly higher (*p* < 0.05) biomass of *L. plantarum* RS5, and lactate concentration was observed in postbiotic metabolite RS5, implying that the antimicrobial activity and lactate production by *L. plantarum* RS5 are growth-associated. Moreover, Chaline et al. [78] also reported a similar observation, whereby the maximum biomass of *L. plantarum* BL011 contributed to the maximum lactic acid production.

## 4. Conclusions

In conclusion, the effects of various combinations of carbon and nitrogen sources on the growth of six *L. plantarum* strains and the respective lactic acid production and bacteriocin inhibitory and antioxidant (reducing power and hydroxyl radical scavenging) activities of their postbiotic metabolites were investigated in this study. UL4 produced the highest viable cell population amongst the *L. plantarum* strains when sucrose as the carbon source and Nucel875 as the nitrogen source were supplemented in the growth medium. The UL4 strain also produced the strongest bacteriocin inhibitory activity when grown in a medium comprising dextrose as the carbon source and FM888 as the nitrogen source. In comparison, the RI11 strain produced the highest lactic acid concentration amongst the six *L. plantarum* strains, with dextrose as the carbon source and Nucel875 MG as the nitrogen source and the highest reducing power of RS5 and TL1 postbiotic metabolites were detected when both strains were grown in an MRS medium. As for the hydroxyl radical scavenging activity, the combination of sucrose and X-SEED KAT induced RG14 to produce the highest hydroxyl radical scavenging activity. The effects of different combinations of carbon and nitrogen sources on the viable cell population of *L. plantarum* strains and the respective functional characteristics of the postbiotic metabolites were strain-dependent, implying a different combination of carbon and nitrogen sources should be optimised subsequently to enhance the specific functional characteristic of the postbiotic metabolites produced by the specific *L. plantarum* strain to warrant vast applications of postbiotic metabolites in respective industries, such as livestock, food, health supplements and medical treatment. The current study also revealed that fermentation media were an important factor that greatly impacted the functionalities of postbiotic metabolites due to the presence of various bioactive compounds that contributed to high antioxidant and antimicrobial properties. 

## Figures and Tables

**Table 1:** Media compositions of formulated media for postbiotic metabolite productions by the six strains of *Lactiplantibacillus plantarum*.

No.	Medium Components (g/L)	MRS	*L. plantarum* Strains					
			RG11	RG14	RI11	RS5	TL1	UL4
1.	Carbon sources (glucose, lactose, sucrose, dextrose)	20	20	20	20	20	20	20
2.	Nitrogen sources (X-SEED KAT, X-SEED Peptone and X-SEED Nucleo Advanced, Nucel875 MG, FM888, FM902)	4	27.84	27.84	27.74	27.84	27.84	36.2
3.	Casein-derived peptone	10						
4.	Meat extract	8						
6.	Sodium acetate	5	5.74	4	5	4.48	3.70	4.88
7.	Tween 80	1	1.12	1	1	1.19	0.76	1.01
8.	Manganese sulphate tetrahydrate	0.04	0.05	0.03	0.04	0.06	0.03	0.04
9.	Magnesium sulphate	0.2			0.2	0.3		
10.	Diammonium hydrogen citrate	2		1.5	2			
11.	Dipotassium hydrogen phosphate	2			2			

Note: MRS medium was used as a control and reference medium to replace the glucose and yeast extract as carbon and nitrogen sources in the respective formulated media for postbiotic metabolite productions by the six strains of *L. plantarum*.

**Table 2:** The best combination of carbon and nitrogen sources induces the highest cell growth for the six strains of *Lactiplantibacillus plantarum*.

*Lactiplantibacillus plantarum* Strains	Carbon Sources	Nitrogen Sources	Viable Cell Number (log cfu/mL)
RG11	Glucose	FM888	9.52 ± 0.03 ^c^
RG14	Sucrose	FM888	9.28 ± 0.03 ^d^
RI11	Sucrose	X-SEED KAT	9.54 ± 0.02 ^c^
RS5	Lactose	X-SEED Nucleo Advanced	9.93 ± 0.04 ^a^
TL1	Dextrose	Nucel875 MG	9.66 ± 0.03 ^b^
UL4	Sucrose	Nucel875 MG	9.98 ± 0.05 ^a^

Note: The values for viable cell number are the mean ± standard deviation (SD), n = 3. Mean ± SD within the same row that do not share a similar superscript are significantly different (*p* < 0.05).

**Table 3:** Correlation between the growth of six *Lactiplantibacillus plantarum* strains and the functional characteristics of postbiotic metabolites.

Concentration	Bacteriocin Inhibitory Activity	Reducing Power	Hydroxyl Radical Scavenging	Lactic Acid
Viable cell growth	0.038 ns	−0.36 ****	−0.35 ****	−0.01 ns
Bacterion inhibitory activity		0.16 *	0.41 ****	0.01 ns
Reducing power			0.39 ****	0.06 ns
Hydroxyl radical scavenging				0.07 ns

Note: * Significant at (*p* < 0.05), **** significant at (*p* < 0.001), and ns, not significant. n = 168 samples per functional characteristic.

## Data Availability

The datasets used and/or analysed during this study are available from the corresponding author upon reasonable request.
